# Book Reviews

**Published:** 1805-03-01

**Authors:** 


### CRITICAL ANALYSIS
### OF THE
### RECENT PUBLICATIONS
### ON THE
### DIFFERENT BRANCHES OF PHYSIC, SURGERY, AND MEDICAL PHILOSOPHY.

---

*Some recent Cases of Small-Pox subsequent to Vaccination; to which are added, Experiments to ascertain the Effects of Vaccinating in the Hand, in imitation of the casual Disease; with Facts and Observations on the Effect of Eruptive Diseases in removing the Security derived from the Cow-Pox.* By W. GOLDSON, Member of the Royal College of Surgeons, London. 8vo. pp. 134.

WHEN we consider the immense progress of vaccination, we shall be surprised that this should be the first serious opposition to one of the greatest novelties that medicine has ever encountered. If we compare it with inoculation, the opposition seems as nothing; if we add to this, the vast proportion of subjects that have been vaccinated, in comparison of those who were inoculated, we shall at once be disposed to respect the good sense of the public, and exalt our security from vaccination.

When inoculation was first introduced, though sanctioned by royal example and the opinion of the most esteemed practitioners, its progress was slow. This was not to be wondered at, because its success was much lessened by the erroneous method of treatment. When the Suttons added to the operation the treatment recommended by Sydenham, the encouragement of the public kept pace with their increased success. Still, however, there were instances in which inoculation, with all these advantages, failed, and few parents or practitioners consented to or undertook the operation without a certain degree of anxiety. The experience of a Dimsdale had also decided that the operation was less successful before a child had arrived at two years of age, till which time a mother could not part with the offspring of her womb without increased solicitude.

At length the genius and perseverance of a Jenner, by turning to a proper use what was before known, obviated all these difficulties, and the public received a boon, the value of which they instantly learned to appreciate. The success bore down all opposition, and the public mind seemed only divided, who should most promote the advantages of so laudable a discovery. In the midst of this security, whilst the friends of humanity were looking forward to the extinction of so dreadful a malady, a new alarm arose; the more serious, as it reduced every thing to uncertainty.

We cannot help lamenting, that the gentleman who first started these doubts was not treated with more delicacy. We are aware too, that it will be said, our Journal has been the vehicle of some severe suggestions. But we trust candour will impute this to our determined impartiality, as we have not been less ready in admitting whatever was offered on either side. And if we thought it our duty to admit the most free discussions from all our correspondents, we shall not be less careful in offering the most impartial review of the work before us.

The preliminary remarks show, that the author is somewhat sore from the treatment he has met; if it produces here and there a little asperity of language, however venial it may be, as we could wish such passages had been omitted, we shall avoid bringing them forward. A much more important remark arises from a quotation from Dr. Jenner's account of the progress of *equination*, viz. that such a process proved "a security from the small-pox for six years, but not for twenty years." We must add, however, that when the small-pox appeared in the last case, it wanted some of the characters of the genuine disease.

The author now begins with a detail of his former cases. As he admits the case of Mr. Grant's child was less prominent in its features than some of the others, we shall dismiss it without those remarks which he has thought proper to subjoin. Nor do we think it necessary to dwell on the report of the French physicians, which proves that vaccination was a security for more than a twelvemonth. Dr. Rollo's cases are next brought forward, with some remarks. These we shall also pass over. Then follow a string of cases communicated by Mr. Crockford, of Lewes in Sussex, from which we are not perfectly aware what inferences our author would draw. Five children in the same family were inoculated at the same time, having been vaccinated at different periods. In all, a local effect was produced; but in one, who stands on the earliest list for vaccination, no constitutional symptoms appeared. He is therefore in a similar predicament with the one whose vaccination is of the latest date. In short, the history of the five Fullers, if it proves any thing, would satisfy us that vaccination is a permanent security from small-pox. Most of the succeeding cases, if they took the small-pox at all, took it about a twelvemonth after vaccination; a circumstance which stands unconfirmed by universal experience, and which is contrary to what our author wishes to establish. However, as every part of the first pamphlet has been already perhaps over-canvassed, we shall forbear to make any other remarks than, that if our author shows a little ill temper in his remarks and quotations, it is not without some provocation. Dr. Rollo's cases are again introduced; and Mr. Goldson seems dissatisfied that the public should suspect so many were inoculated, when by far the greater part were only exposed to the effluvia. After this, we have the cases of the Vaccine Institution, at which equal dissatisfaction is expressed, that they were only inoculated, and not exposed. His reasoning on this subject is to us not perfectly intelligible, nor which way the possibility of receiving casual cow-pox more than once is connected with this part of the question.

The

The author now enters on some recent cases which have occurred at Portsmouth and other places. These are related with the same apparent accuracy and candour as in his former pamphlet. No. I. as he justly observes, was not varicella; neither, we may add, was it small-pox. From inoculation we sometimes have eruptions which do not maturate, but die away on the 5th day with a warty scurf on the apex; but such is never the case in casual small-pox, which was all the patient was subjected to. No. II. is liable to the same objection; in casual small-pox the eruptions never die away on the 5th day. We wish the case inoculated from this subject had been described more fully. However, we shall for the present admit that small-pox was produced by inoculating from an unsatisfactory case. No. III. is in all material points similar to the former. No. IV. is a communication from Mr. Dunning, of Plymouth, giving two similar cases to those above stated by the author of this paper. One was from casual exposure to, the other from inoculation of small-pox, two years and a half after vaccination. Mr. Dunning's inferences from the cases are highly favourable to vaccination. Mr. Goldson thinks differently. It is therefore with particular pleasure we see these gentlemen conduct their correspondence with urbanity on both sides.

No, V. and VI. Mr. Goldson now rests his strong arguments on the Fulwood's Rents cases: as these are admitted to be variolous by most of the friends of vaccination, and as all the particulars have been repeatedly before the public, we shall for the present dismiss them. For other reasons we shall pass over Mr. Goldson's remarks on the subject.

No. VII. of Capt. Simmons's daughter, who was inoculated with small-pox three years after vaccination. The effect was very well worth remarking: the arm, for the first four days, inflamed rapidly; after this, seemed to subside; but on the 8th day all the phenomena attending inoculation in the usual way occurred. No. VIII. is related so loosely, (and without the mentioning any authorities) that we cannot take any notice of it.

No. IX. and X. are two cases of Mr. Bowen, of Harrow. We wish these, like Mr. Dunning's, had been given in the words of the relator, as we have heard them stated differently from the account here given. This account is also extremely imperfect, admitting that it no way deviates from truth. No mention is made of the period between inoculation and the commencement of fever.

Having dismissed his cases, Mr. Goldson now enters into a very useful inquiry, in which we sincerely wish his antagonists had anticipated him. It consists principally of observations on what was called a hybrid disease in the early period of vaccination at the Small-pox Hospital. From these, it appears probable that the constitution may in some instances be subject to an eruption from small-pox after vaccination, and that this eruption shall be found to preserve its own form, which is not only milder, but in other respects different from genuine small-pox. We regret with the author thor and Dr. Willan, that this inquiry was not pursued further at the hospital, but doubt not that Mr. Goldson's cases will bring the subject fully under discussion. Some remarks follow on chicken-pox, in which an attempt is made to distinguish it from small-pox. But no external marks are, in our opinion, at all times sufficient to distinguish mild small-pox from severe chicken-pox. The observation that the horny eruptions which have occurred from small-pox after vaccination is different from varicella, is very just.

Our readers will perceive, with pleasure, a prospect of reconciling Mr. Goldson to vaccination. From the above extracts and remarks it will appear, that of the number vaccinated, which is now almost beyond calculation, only a few have shown any symptoms which could arise from small-pox infection, either by inoculation or exposure. Of these, only a single instance or two in the same family had a legitimate small-pox eruption. The rest were cases so different from the true characters of that poison, and generally so similar to each other, that Mr. G. seems disposed to believe vaccination might have been cause of the new character assumed by the disease. But we must observe, contrary to Mr. G's remark, that in the earlier accounts of small-pox inoculation, Mr. G. will find much heavier accusations against that practice, and, as we observed in the beginning, vaccination has been almost universal, whereas inoculation was at first very much confined. Those opposition pamphlets (to inoculation) are now almost forgotten, but, *litera scripta manet*; and whoever will consult the collections of the curious will soon be convinced how much more inoculation had to encounter than vaccination. Still, however, two questions remain undecided; and, for the inquiry into the latter, the public have to thank Mr. Goldson. The first question, Does vaccination ever produce an eruptive disease? The 2d, What is this disease which sometimes occurs after vaccination, capable of producing by insertion a legitimate small-pox, but has itself been uniform in its mildness, and in its character always different from the variolous? In most we believe, if not all the cases, except Fulwood's Rents, the pustules died away prematurely; and even in those cases the progress of the disease was not strictly that of casual small-pox. Yet, that it was of variolous origin, and that it was capable of perpetuating variola, we are not sceptical enough to doubt.

We have next a case of small-pox eight years after casual vaccination. We much regret that this is not related by any medical man who saw the progress of the disease. This case is, however, an awkward introduction to Mr. Goldson's proposal in the next chapter, of vaccinating the hand instead of the arm, in order to imitate the casual cow-pox. We have no doubt that he has seen a difference between the pustule or vesicle in the arm and the hand. The thickness of the cuticle is sufficient to produce this difference, and often does produce it, even in casual small-pox. To make his experiments complete, Mr. G. must vaccinate the arm from the cow.

Next follows a chapter of "facts and observations, submitted to impartial impartial investigation." The fact mentioned (page 114) is worth relating, and to the friends of vaccination may prove very useful. Those who think the observations worth perusing must consult the work, as we know not how to analyse or compress them.

Mr. G. next offers the result of observations on the effect of eruptive diseases, in removing the security derived from cow-pox. This is a question we think few people will treat as he proposes. However, every fact has its importance.

The work concludes with some doubts resulting from what the author has seen, and the train of thinking it has produced. We wish the whole had been written with the same temper. We are, however, pleased to see a postscript subjoined, by which it appears, that, like a true Englishman, Mr. Goldson will not permit other people to abuse vaccination, though he may take that liberty himself. In short, if our author is satisfied with imitating the casual cow-pox by vaccinating the hand, we are ready to join hands with him; and if ever, like us, he should prefer the arm, we shall be ready to embrace a proselyte. At all events, we think science much indebted to him for calling our attention to subjects which would otherwise have been overlooked, if not concealed with an unnecessary, we may add an unbecoming, industry.

---

*Outlines of a Plan, calculated to put a Stop to the Progress of the malignant Contagion, which rages on the Shores of the Mediterranean, if, notwithstanding every Precaution to the contrary, it should unfortunately make its Way into this Country; by* Richard Pearson, M.D. Octavo, pp. 27. London, 1804.

The author does not claim the merit of originality in any of the means he recommends, but he endeavours to digest them into a comprehensive system or general plan.

It embraces *Quarantine*, both of goods and persons, strictly enforced; this however being insufficient alone, Dr. P. advises "other precautionary measures to be joined with it; that if, notwithstanding the utmost vigilance to the contrary, the contagion should get ingress into this country, it may be immediately detected and suppressed.

"For the accomplishment of an object of so much moment, it is proposed that Committees of Health be established in all the principal sea-ports throughout the kingdom. They should consist of an adequate number of physicians and surgeons, assisted, according to circumstances, by the magistrates and clergy. Their business should be to examine into the health of the sea-port towns in which they reside; and whenever a febrile disorder breaks out, that seizes in succession three or four individuals residing under the same roof, or having had recent communication with each other; that is rapid in its course, and accompanied with symptoms of unusual malignancy, immediately to resort to the system of *separation, fumigation, &c.* strict orders being at the same time given, to burn burn all the bedding and clothes that had been used by the sick; and the house or houses in which such a disorder should occur should be thoroughly fumigated, white-washed, and cleansed. These precautions are the more necessary, inasmuch as the plague, in some of its most fatal forms, and especially on its first breaking out, is not marked by its characteristic eruptions.

"In like manner, there should be instituted a General Board of Health in London, not only as the capital of the united kingdom, and consequently maintaining a frequent communication with every other part, but likewise as the largest port-town. This General Board should use the same vigilance in their inquiries into the health of the port and city of London, as the provincial committees in their respective towns; and should resort to the same means of suppression, in case of a strong suspicion, or actual proofs of infection. All these committees should keep a regular journal of their proceedings, and transmit to government a statement of such proceedings as often as shall appear necessary. It would further be desirable that the General Board of Health should draw up a set of Instructions for the use of the before-mentioned committees, and of medical practitioners in all parts of the kingdom.

"In the above proposals for instituting Committees of Health, I have merely hinted at the measures of *separation, fumigation, &c.* without entering into a description of the manner of doing them; these matters having been so amply detailed in various tracts which have been recently published. Indeed, the directions in this respect will constitute a principal part of the business of the before-mentioned committees; and should be minutely set forth in the Instructions published by the General Board of Health in the metropolis.

"Relative to this business, I shall only remark, that the building or buildings hired as a receiving house or houses, (according to the number of the infected) should be situated in open, and, if possible, elevated situations at the extremity of the town, or rather entirely out of it; and that in the event of a sea-port town or inland-port town becoming the seat of infection, *floating lazarettos* (as recommended by Dr. Blane) seem preferable to lazarettos within land. As in the case of the above-mentioned receiving-houses there should be three distinct buildings (unless one building be sufficiently large; in which case, there should be three distinct ranges of apartments) for the *sick*, the *convalescent*, and the *suspected;* so, in the case of floating lazarettos, there should be as many separate vessels for these three different classes of persons."

Dr. P. then proceeds to obviate some objections, and detail some particulars respecting the plague of London and the causes of its extensive propagation, which he doubts not, may, in future, be prevented.

---

*An Account of two Cases of Gout, which terminated in Death, in Consequence of the external Use of Ice and cold Water.* By A. EDLIN. 12mo. pp. 24.

Mr. BAKER, a surgeon of eminence at Uxbridge, was attacked with regular gout on the feet, on the 22d of July last, which continued nearly stationary for three days, unattended with fever, or any material inconvenience. The fourth night he passed restless and unquiet, the pain and inflammation increasing, but still confined to the feet. At this time Mr. B. who had received a strong and favourable impression of the excellence of cold applications, by perusing the several communications on the subject, with which our correspondent, Dr. Kinglake, has favoured this Journal, resolved to make the experiment himself, and accordingly applied first cold air, then sponging with cold water, and lastly cloths dipped in ice water; with manifest relief of the pain and inflammation. About five hours afterwards the pain was quite gone, and the pulse 78. On getting out of bed, however, a fainting fit came on, which was removed, and he took some breakfast. A slight inflammation was observed in the knees, which was removed by the iced water. Four hours afterwards an attack of alarming symptoms occurred, which is thus related by Mr. Edlin. "At one o'clock in the afternoon I was suddenly called to him again, and found the whole family in a state of the utmost consternation and alarm; on entering his chamber, I found him lying on his back, with a difficult, hurried respiration; his extremities were cold, his pulse quick, fluttering, and intermitting; he complained of a palpitation of the heart, and an icy coldness in the stomach: he had vomited several times, and a cold sweat had broke out on the skin. Some warm brandy and water was immediately given him, and in the mean time a draught with æther, camphorated julep, and opiate confection was prepared; bladders of hot water were now applied to the region of the stomach, the knees, the hands and the feet, in the hope of inducing a return of the disease to the extremities; but, after waiting some time, and no relief being obtained, I gave him the same draught again, with the addition of a scruple of musk. The pain and oppression of the breath now began to decline, and a gentle perspiration broke out on the skin, which continued the remainder of the afternoon."

In the evening, with the advice of Dr. Haworth, the extremities were fomented, and some sleep was obtained; and for two entire days no great change of symptoms occurred: but on the next day, (three entire days from the first cold application) another shivering fit and attack of gout in the stomach took place; and, after lingering three days longer, the patient died.

The above is the principal case here related, in which the writer was an eye witness; the other is less circumstantial, as it happened thirty years ago, and is given only on hearsay evidence, though apparently unimpeachable.

Mr.

Mr. Edlin makes no comment on this case, considering it as being of itself a most irrefragable argument against Dr. Kinglake's theory and plan of practice. But the testimony of facts is not the only attack which the author makes on Dr. K's system. In endeavouring to dissuade his friend from the rash application of cold, which brought on so calamitous a termination of the disease, Mr. Edlin, it seems, insinuated a doubt of the veracity of the statement made by Dr. Kinglake, which doubt he has not hesitated here to render public. "I hinted," Mr. E. says, "that it was possible these might be fictitious cases, composed for the purpose of explaining a particular theory; and observed, that I was sorry to see him misled by so shallow a performance. However, as I found that he was not disposed to give up either his opinion or his practice, I took my leave, at the same time observing, that I was afraid he would not be convinced till he had drove the complaint to his stomach." Common liberality required of Mr. E. something more than an unsupported insinuation to justify so serious an accusation as that of *case-coining;* and this circumstance completely removes the flimsy veil of candour which the author affects to throw over his narration.

---

*Remarks on the Constitution of the Medical Department of the British Army; with a Detail of Hospital Management, and an Appendix, attempting to explain the Action of Causes in producing Fevers, and the Operation of Remedies in effecting the Cure. By* Robert Jackson, M. D. 8vo. pp. 351; with the necessary Tables. London, 1803.

The Constitution of the medical department occupies about half the volume, and the observations contained in it, we understand, have been duly attended to by the Inspector General of Hospitals. This part concludes with the account of the causes which induced Dr. J. to resign his situation at the Depôt in the Isle of Wight. In this statement the author's modesty induced him to omit several passages in the official documents, which, though highly gratifying to his own feelings, were not essential to his work. He afterwards, however, thought himself compelled, in his own justification, to publish these omitted passages in a letter addressed to the Editor of the Edinburgh Review, where he considered himself as traduced and misrepresented. We are sensible that the Commander in-Chief had sufficient cause to express his regret, in the letter in which he accepts Dr. J's resignation, at the loss of the services of so valuable a medical officer; the best, if not the only reparation for such a loss, was the work before us.

It is an attempt to transfer Dr. J's knowledge and experience to the whole medical world; which we deem impossible to be done in a short Essay, or in less time than half a century. The well-educated practitioner, however, cannot fail to profit materially by the hints and observations contained in this short outline of the treatment of Fever.

S 2 We

We think it right, before we proceed to analyse the parts of which it consists to observe, that Dr. J. has devoted his whole medical life to the study and investigation of fever; that he has seen this order of diseases longer and more frequently, in a greater variety of climates and under a greater variety of circumstances, than almost any other physician; that, notwithstanding this, there are several classes of febrile patients who have not or could not have fallen under his notice. His patients have generally been *young robust men* in the army or navy. Those species of fever which have principally engaged his attention, were of the most concentrated virulence, and malignant kinds; for he appears to have believed, that if these could be brought under medical controul, milder cases would follow in the train. We must also observe, that Dr. J. generally practised under circumstances which gave him a fair scope for his ability. He had not the weakness of parents, the negligence of nurses, nor the perverseness of patients to contend with. We may presume that his abilities had fair play.—These observations appeared necessary to be premised, in order to place the author and his readers on their just level, so that the one may not be condemned for omitting what he never thought of saying, nor the others disappointed by expecting what it was impossible for the author to say with propriety.

The first part of this Essay is directed to the operation of the causes which produce fever. This subject has puzzled theorists for two thousand years, and probably will exercise their ingenuity for the succeeding two thousand. Dr. J. deemed it necessary to say something on that part of the subject; and we have no doubt that a large majority of his readers will be less pleased with his theory than with his practice. It seems, however, necessary for every author to explain the *terms* he uses; and this can hardly be done so well as in a statement of his system. Dr. J. does not adopt the theory of Cullen, Brown, or Darwin; but he appears to prefer the latter. The principal new term which he has introduced, is, that which denotes the balance and regular movements of all the functions during health, and the derangement of this regularity produced by the causes of fever. As the manner in which these causes produce their effect will probably never be understood, medical writers ever have been, and perhaps ever will be, in the habit of expressing these states of the human frame by some figurative term, which, if happily adapted by a popular author, becomes general among his pupils or followers. A musician would express them by the terms *harmony and discord*; an astronomer, by the terms, *regular and perturbed motions*; a poet, by the terms, *regular and disjointed rhythm*. Dr. Jackson has chosen the latter expression: so that "harmonious rhythm," "natural rhythm," "disturbed or perverted rhymth," occur frequently in this Essay. As it is the chief object of our Journal to convey *practical* information to our readers, we shall refer such as desire to see Dr. J's theoretical opinions to the work itself, and only only select a few passages from the practical parts. D. J's principal remedies are, bleeding, emetics, bathing, gestation, bark, wine, opium, and blisters. He appears to value the first of these far more highly than any of his cotemporaries. It is, indeed, generally considered as inadmissible in malignant fevers; but Dr. J. deems it on many occasions to be an essential part of the preparation for his curative means. He says,

"Where the condition is present, which warrants the attempt of arresting the disordered rhythm of movement, previous to the application of causes calculated to excite motions analogous to those of health, the means differ, according to the circumstances of the case and condition of the subject. Of preliminary remedies employed in this view, bleeding and emetics are two of the most powerfully and most generally applicable. When the diseased motions have been arrested, or subverted by the employment of these or other means, the pure air of the atmosphere, the universal and common stimulant of life, is often sufficient to solicit the organic structure to resume its natural action. But, where the power of this fails, or is supposed not to be sufficient to effect the purpose, others, which are of greater force, or which are capable of more direct management, are called in aid. Of these, bleeding, alternate warm and cold bathing, are among the most powerful; and as the manner of using these, and some other remedies, may not have been fully explained by the author in his former publications, he will now attempt to define such circumstances as he had then overlooked altogether, or touched in a hasty manner.

"Bleeding. Bleeding has, perhaps, experienced more revolutions of reputation as a remedy in the cure of fever, than any other remedy which has been employed by physicians. In some ages, and in some countries, it has been considered as the cardinal hinge of medical means; in others it has been, and by some it is even now, regarded with abhorrence, considered as a practice most certainly paving the way for destruction. Such opposite opinions can scarcely be accounted for. They serve to bring disgrace on the medical profession; for, as diseases are fundamentally the same now, which they were fifteen hundred or one hundred years ago, Galen and Sydenham must either be supposed to have been singularly deceived, in reporting so favourably of the effects of this remedy; or we must ourselves be supposed to be precipitate, in condemning, as hurtful, the use of means which they found to be so beneficial. Galen was a man of great knowledge; Sydenham was a man of great candour. If the one reasoned well, the other reported truly. It may be alleged, that their opinions concerning causes biassed their judgment, and led them into error. From such opinions, the present times are not exempted. If Galen was led to the use of the lancet, by a preconceived opinion of plethora, we are deterred from it by a be-

 lief lief in debility,—an opinion not resting on better foundations than that of plethora, viz. both effects of febrile action,—not causes.

"It must not, therefore, be understood, that BLEEDING is considered by the author as a debilitating process. Its effects are stimulative, relatively according to the circumstances of the subject: and they are extensive, for they are felt in all parts of the circulating system, and consequently, through the whole extent of the animated machine. The abstraction of blood, by its express effect, diminishes the quantity of a body to be moved; and thereby increases the power of the mover: it thus facilitates motion; but this is not all. The diminution of the quantity of blood, and change of movement in consequence of such diminution, is in some manner productive of change of condition at the sources of life: motion is affected,—changed, even suspended; diseased motions are arrested; an opportunity is thereby furnished for the more effective action of those powers, which are provided and expressly calculated for the stimulation of the due action of health. Bleeding, as it is the most manageable power, so it possesses the most absolute influence over animal movement, either as directly effective of a final purpose, or as preparatory to the action of other means necessary to insure the final purpose.

"Among the general circumstances, which influence medical practitioners in the use of bleeding, may be reckoned age and sex. It has been already observed that bleeding is less necessary, where there already exists susceptibility of impression. On this ground, it is less necessary in tender years, and in youth, than in grown persons, or in old persons; less necessary in females than in males. It is also less necessary in persons of a soft, thin skin, a lax and delicate fibre, than in the opposite circumstances. It also has appeared to be less necessary among the inhabitants of champaign, fertile and moist countries, than among the inhabitants of hilly, barren and dry districts. It is less necessary, for the most part, among the rich, luxurious and enfeebled classes of man, with their excesses of sensibility of mind and body, than among the poor, temperate, and hardy rustic;—less necessary in summer, unless in very hot and very dry weather, than in spring; and less necessary in autumn, than in winter.—These are general circumstances, which have, or which ought to have, some influence in forming an opinion concerning the propriety of bleeding;—knowledge of them may often be useful, in directing the proper measure in point of quantity. The particular circumstances belong to particular subjects, under particular forms of malady.

"In the earlier stage of fever, where the pulse, in its motion, is irregular in time and force; in its action, quick, hard, irritated, and labouring to overcome a resistance; or where it is regular in time, but sluggish in action,—oppressed, and without energy, that is, without freedom of expansion and celerity of contraction, the effects of bleeding are equally beneficial, though the conditions be seemingly different. The circumstances, which accompany accompany these opposite states of the pulse, are also different and peculiar. In one, the respiration is quick and hurried,—and the breath is hot; in the other, respiration is oppressed, interrupted with sighing, or connected with a difficulty of expanding the chest,—as if from want of power,—without interruption from pain. The pain of the head, in the one, is rending and tensive, with strong pulsation of the carotid and temporal arteries,—and with great heat on the forehead; in the other it is heavy and dull, with stupor and inability of commanding thought,—with a clammy, and sometimes a cold forehead. The countenance, in the one, is agitated and confused, with marks of agitation, expressive of pain,—sometimes threatning and grim; in the other it is heavy and torpid, sometimes dark, bloated, and inanimate. In the one the skin is thick, the surface dry and distended; in the other, damp and greasy, without that activity of life, which occurs in natural perspiration. In one the heat is deep, concentrated rather than superficial,—ardent, rather warm; in the other it is often below the just point of heat in health, but its impression is different from that which the heat of a healthy body imparts,—known by the sensation to persons of experience, but not easily described. In one, the pains in the back and limbs are severe, and all the joints seem as if they were broken and unhinged. In the other, there is torpor, — an inability of producing action of effect rather than any excess of pain. In both, the secretions are suspended with a sense of fulness and tumult, or with marks of bloated stagnation and inability. These conditions are different externally; but they are often only parts in the same chain, under different circumstances of expression;—they are under the influence of the same remedy in the processes of cure.

" In the other condition of general fever, where the pulse is open and unconfined, expanding, or as it were rising to the surface, the heat superficial, warm, rather than ardent or pungent; the skin soft, thin, giving an impression of buoyancy and elasticity; the countenance clear; pains, if they exist, of a sharp nature, flying through different parts, but not connected with sensations of weight and oppression, bleeding is not by any means necessary. Such form of disease generally terminates by regular crisis on or before the seventh day, when left to itself, or treated with gentle remedies. If it be determined to cut short its course by forcible means, the abstraction of a small quantity of blood will be serviceable; for by increasing the susceptibility of impression, the means afterwards employed to excite new motions will act with more certain effect. The remedy however is not indispensable; and, where useful, the quantity to be taken away is limited to a measure not exceeding twenty ounces. Such form of disease is more common in mild, moist, and warm weather, among persons not exposed to causes of unusual violence, as excess of cold or heat; persons of soft skins and delicate frames; as females and persons in the higher ranks of life.

"In fevers of the genuine periodic form, bleeding is a remedy for which there rarely is occasion. Where the intermissions, or remissions are distinct, the paroxysms terminating by copious perspirations, by copious vomitings and bilious evacuations, plethora not existing, and the fibre seeming to possess sensibility to impression, bleeding is by no means necessary: yet, in certain conditions of fever, radically periodic, but, from force of cause or circumstance of subject, not assuming their genuine form, it is often not only useful but indispensable. Where the pulse, in the state of remission, conveys an idea of hardness, confinement, or want of expansion, particularly where connected with a clouded aspect and defective secretions, bleeding is usually followed by distinct intermission; and the disease enters into the common road of cure. Further, in instances of endemic fever, fundamentally periodical, but connected with a dry, thick, and torpid skin, a bloated countenance, suspended secretions, a sluggish and oppressed movement, or an irritated movement without productive effect, bleeding is indispensable; for it is the only remedy with which we are acquainted, capable with any certainty of removing the mask which covers the genuine expression of the disease. It effects a change in the circumstances of movement; the susceptibility of impression is restored; the disease appears in its own character, and yields to ordinary remedies. It is probable, even more than probable, that the fever, which has committed such devastation in America for some years past, was only conditionally malignant, masked by circumstances of a more general or local nature, so as not to appear in its real character. The circumstances of its history prove it incontestably to be endemic; and there is good reason to believe, that bleeding, to the extent here recommended, could have brought it to assume a remitting form, and consequently would have brought it within the reach of common means of cure. Such, at least, was the effect of the treatment proposed in the concentrated endemic of St. Domingo, with which the fever of America seems to have a great resemblance.

"Besides the above rules for regulating the use of bleeding, in the early stage of periodic fevers, there sometimes occurs a condition in these diseasess, of very difficult discrimination, but of very great importance to be correctly known. In certain forms of this disease, of a latent malignity of character, the paroxysms sometimes come on unexpectedly with stupor, suspension of sense and motion, or with the evident oppression of some important organ, the head or lungs. Sometimes, there is no visible accompanying tumult in the circulating system; the suspension continues for a given period, and ceases without a visible effect. Sometimes, there is an evident tumult in the circulation,—a marked determination to particular organs:—Evacuation or effusion into a cavity—the head or chest,—is the consequence. In the first, bleeding is not necessary; in the last, prompt and copious bleeding, so as to make a decided change in the existing circumstances of the the case, is the only means which can save life. To discriminate such conditions, is not always easy; and they are sometimes, perhaps, in some degree, complicated. In the one case, the neglect of bleeding is destruction; in the other, though it may not be necessary, it is not injurious. In all cases of doubt, it will therefore be adviseable to adopt the safer measure, taking away blood where function is merely suspended from a caprice in movement, rather than neglect it, where function is oppressed by quantity, endangering suffocation.

"The fever, which arises from a contagious source, does not appear precisely in the same form, or ordinarily tend to termination by a similar product, with that which arises from endemic causes. The effect is not so strongly and permanently manifested in the grosser circle of circulation. The natural secretions are not usually so completely suspended, during the continuance; and the termination is not so uniformly marked, by an increased evacuation, or distinct crisis. The action of the cause of this disease manifests itself, in the first instance, in the stomach; the functions of which, it in some manner impairs. Its first manifest action, as a febrile irritation, when the cause is not controuled by counteracting circumstances, is perceiveable in the surface of the body, the conditions of which are generally changed in one form or other, either as expressed by an apparent increase, or by an apparent defect of action. The skin and stomach have a radical sympathy; and it is principally upon the stomach and skin, and their connexions, that the cause of contagious fever exerts its force. The skin, in one form of this disease, is hot, sensible, often painful to the touch; it burns, but without strong heat; the face flushes, as from wine or heated air; the eyes appear red; they are hot and painful, sometimes glisten brilliantly; the pains of the head are often severe, sharp, even shooting, but irregular and inconstant; pains fly rapidly through the limbs in explosions; the pulse varies; sometimes it is frequent and quick, even full and open; it rarely expresses a sense of struggling motion as if from resistance, so common in some other fevers: sometimes it is little changed in point of time; but it always expresses some change in condition, though it be not describable in words. There is, upon the whole, something peculiar in the sensation; the motions are of a lighter kind than in endemic fever, and the observer gets an impression, that a different circle of movement is affected; it is not possible to say in what manner. Bleeding is rarely necessary in this fever. In the more concentrated forms which attack with a stupor like apoplexy, or which are accompanied with motions of unusual irritation, it may sometimes be useful as the first remedy. But unless where this is the case, an emetic is more properly first, in order of time, to commence the cure of the contagious fever. It does not indeed often happen, that bleeding is a remedy absolutely necessary in contagious fever; yet it is sometimes useful, and it is by no means destructive, as practitioners seem generally to believe. Wherever the the susceptibility of impression is deficient, particularly where such deficiency is connected with an appearance of fulness, a dry and constricted surface, bleeding may not only be safely employed, but, by restoring susceptibility and all its consequences, it eminently tends to render the other means safe and effectual of their purpose. That it should be so seldom necessary, seems to depend upon the mode of action of the morbid cause, which rarely has an operation calculated to absorb the sensibility of the system.

"In the opposite condition of contagious fever, where the countenance is sallow and dull, the skin greasy, damp, dirty, and cold; the eye white, and idiot-like, with a general look of despondence and want of animation; where there are no marks of oppression of organs, or signs of general plethora, — a condition frequent in damp weather, crouded and unventilated places, bleeding is not useful; — it is not certain how far it is safe. In cases of increased mobility, with tremors, faintings, a skin open and free from constriction, it is not only unnecessary, but evidently hurtful.

"Bleeding is useful, is indeed a remedy of the first value in the purely rheumatic fever, particularly as preparatory to the other means of cure, which consist in the various processes and alternations of warm and cold bathing. Here, the quantity must be measured by effect under the operation,—modified according to the circumstances of the case, but carried to the point to insure the effectual operation of the other remedies. It is scarcely necessary to say, that the bleeding must generally be a large one.

( To be continued. )

